# A HER2‐targeted Antibody‐Drug Conjugate, RC48‐ADC, Exerted Promising Antitumor Efficacy and Safety with Intravesical Instillation in Preclinical Models of Bladder Cancer

**DOI:** 10.1002/advs.202302377

**Published:** 2023-10-12

**Authors:** Xuwei Hong, Xu Chen, Hongjin Wang, Qingchun Xu, Kanghua Xiao, Yuanfeng Zhang, Zepai Chi, Yeqing Liu, Guangyao Liu, Hong Li, Jianmin Fang, Tianxin Lin, Yonghai Zhang

**Affiliations:** ^1^ Department of Urology Shantou Central Hospital Shantou Guangdong 515031 P. R. China; ^2^ Department of Urology Sun Yat‐Sen Memorial Hospital Sun Yat‐Sen University Guangzhou Guangdong 510120 P. R. China; ^3^ Guangdong Provincial Key Laboratory of Malignant Tumor Epigenetics and Gene Regulation Sun Yat‐Sen Memorial Hospital Sun Yat‐Sen University Guangzhou Guangdong 510120 P. R. China; ^4^ Department of Pathology Sun Yat‐Sen Memorial Hospital Sun Yat‐Sen University Guangzhou Guangdong 510120 P. R. China; ^5^ School of Medicine South China University of Technology Guangzhou Guangdong 510120 P. R. China; ^6^ BioMed Laboratory Guangzhou Jingke Biotech Group Guangzhou Guangdong 510120 P. R. China; ^7^ RemeGen Ltd. Yantai Shandong 264006 P. R. China; ^8^ School of Life Science and Technology Tongji University Shanghai 200092 P. R. China; ^9^ Guangdong Provincial Clinical Research Centre for Urological Diseases Guangzhou Guangdong 510120 P. R. China

**Keywords:** antitumor activity, HER2, intravesical instillation, non‐muscle‐invasive bladder cancer, RC48‐ADC

## Abstract

More than half of non‐muscle‐invasive bladder cancer (NMIBC) patients eventually relapse even if treated with surgery and BCG without optional bladder‐preserving therapy. This study aims to investigate the antitumor activity and safety of a HER2‐targeted antibody‐drug conjugate, RC48‐ADC, intravesical instillation for NMIBC treatment. In this preclinical study, it is revealed that human epidermal growth factor receptor 2 (HER2) expression scores of 1+, 2+, and 3+ are recorded for 16.7%, 56.2%, and 14.6% of NMIBC cases. The antitumor effect of RC48‐ADC is positively correlated with HER2 expression in bladder cancer (BCa) cell lines and organoid models. Furthermore, RC48‐ADC is revealed to exert its antitumor effect by inducing G2/M arrest and caspase‐dependent apoptosis. In an orthotopic BCa model, tumor growth is significantly inhibited by intravesical instillation of RC48‐ADC versus disitamab, monomethyl auristatin E, epirubicin, or phosphate‐buffered saline control. The potential toxicity of intravesical RC48‐ADC is also assessed by dose escalation in normal nude mice and revealed that administration of RC48‐ADC by intravesical instillation is safe within the range of effective therapeutic doses. Taken together, RC48‐ADC demonstrates promising antitumor effects and safety with intravesical administration in multiple preclinical models. These findings provide a rational for clinical trials of intravesical RC48‐ADC in NMIBC patients.

## Graphical abstract

Intravesical administration of RC48‐ADC demonstrates promising antitumor efficacy in human bladder cancer cells expressing HER2 in vivo, and it is also well tolerated with no detectable local or systemic toxicities at the therapeutic dose. These preclinical findings provide evidence to support further clinical trials of intravesical RC48‐ADC in patients with non‐muscle‐invasive bladder cancer to improve therapeutic outcome.

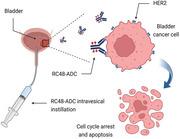

## 1 Introduction

Bladder cancer (BCa) is one of the most common malignancies of the genitourinary tract, with approximately 573 000 new cases and 212 000 deaths per year worldwide.^[^ ^1^, ^2^ ^]^ It is a clinically heterogeneous disease that is divided into non‐muscle‐invasive BCa (NMIBC) and muscle‐invasive BCa (MIBC) according to the depth of tumor invasion.^[^ ^3^ ^]^ Novel urinary assays facilitate the detection of early stage tumors, and 70–80% of new cases are diagnosed as NMIBC.^[^ ^4^ ^]^ However, 55–85% of patients with NMIBC experience recurrence within 5 years despite treatment by transurethral resection of bladder tumor (TURBT) combined with intravesical Bacillus Calmette‐Guerin (BCG) or chemotherapy.^[^ ^5^ ^]^ The multifocality of tumor and residual tumor cells due to incomplete resection may explain the high recurrence rate of BCa. Intravesical instillation therapy kills tumor cells directly or induces a local immune response by instilling the bladder with cytotoxic or immune agents to reduce the risk of tumor recurrence and progression.^[^ ^6^ ^]^ However, almost half of patients eventually relapse even if treated with BCG without optional bladder‐preserving therapy.^[^ ^7^ ^]^

In recent years, antibody‐drug conjugates (ADCs) have shown compelling efficacy and survival benefits versus chemotherapy and immunotherapy in patients with locally advanced or metastatic BCa.^[^ ^8^, ^9^ ^]^ Several clinical studies have shown that ADCs, such as enfortumab vedotin (NECTIN4‐ADC),^[^ ^10^ ^]^ disitamab vedotin (HER2‐ADC),^[^ ^11^ ^]^ and sacituzumab govitecan (TROP2‐ADC),^[^ ^12^ ^]^ significantly improve the disease control rate of advanced BCa and prolong progression‐free survival (PFS) and overall survival (OS) of patients. Despite the satisfactory efficacy and tolerance of ADCs for advanced BCa, the high incidence of treatment‐related adverse events (TRAEs) (94–100%) caused by systemic administration cannot be ignored; the incidence of TRAEs of grade ≥3 is 54–58.1%.^[^ ^13^ ^]^ Considering the promising efficacy of ADCs in the treatment of advanced BCa, the timing of ADCs treatment is expected to move forward, especially in the treatment of high‐risk NMIBC. For NMIBC, intravesical instillation could enable direct exposure of drugs to BCa cells with reduced systemic exposure and adverse events compared with systemic administration.

Human epidermal growth factor receptor 2 (HER2) has been proven to be overexpressed in many tumors, including BCa, gastric cancer, and breast cancer. It is known to contribute to promoting cell proliferation and is associated with aggressive growth and poor clinical outcomes of patients.^[^ ^14^, ^15^ ^]^ The positive rate of HER2 in BCa reported in the literature varies greatly, ranging from 8% to 70%.^[^ ^16^, ^17^, ^18^ ^]^ Recently, a large sample study involving 37 992 patients showed that the incidence of HER2 overexpression in BCa (12.4%) was even higher than that in breast cancer (10.5%).^[^ ^19^ ^]^ Furthermore, a previous study found that HER2 was a biomarker for predicting the prognosis of NMIBC, which helps identify patients with a high risk of progression and recurrence.^[^ ^20^ ^]^ Collectively, these findings indicate that intravesical instillation of HER2‐targeted ADCs is a reasonable choice for patients with HER2‐positive high‐risk NMIBC.

RC48‐ADC is a novel HER2‐targeting antibody‐drug conjugate (ADC) that selectively delivers the cytotoxic agent monomethyl auristatin E (MMAE) to HER2‐positive tumor cells. It was well tolerated and showed promising efficacy in HER2‐positive advanced BCa in previous clinical trials.^[^ ^11^, ^21^, ^22^ ^]^ However, the antitumor activity and safety of RC48‐ADC intravesical instillation for the treatment of NMIBC remain unknown. In this preclinical study, the expression of HER2 was measured in NMIBC tumor tissues and various BCa cell lines. The effects of RC48‐ADC on cell viability, cell cycle arrest, and apoptosis were investigated in vitro. The antitumor efficacy and potential toxicity of RC48‐ADC were evaluated in an orthotopic mouse BCa model with intravesical instillation in vivo.

## 2 Results

### 2.1 HER2 is Highly Expressed in NMIBC and BCa Cell Lines

**Figure 1:**
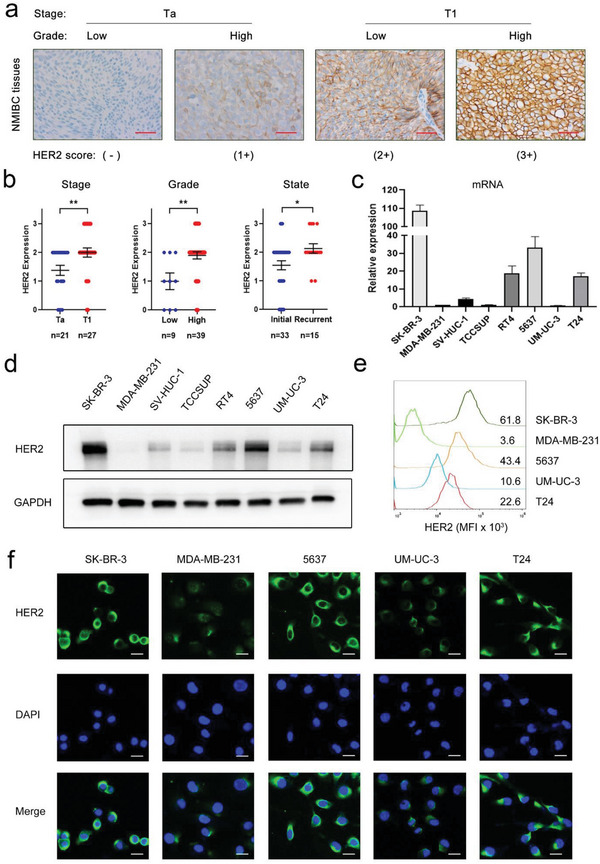
HER2 is highly expressed in NMIBC and some BCa cell lines. a) Representative images of the cell surface HER2 staining in NMIBC tissues according to American Society of Clinical Oncology (ASCO) guidelines. Scale bars: 50 µm (red). b) HER2 expression score on IHC with respect to pathological tumor (pT) stage, histological grade, and disease state. Relative gene expression of HER2 measured by c) qRT‒PCR and protein expression measured by (d) Western blotting in BCa cell lines and breast cancer cell lines. SK‐BR‐3 [HER2 high] and MDA‐MB‐231 [HER2 low] cells were chosen as references. e) FCM and (f) IF staining verified the expression of HER2 in 5637, T24, UM‐UC‐3, and breast cancer cell controls. Scale bars: 20 µm (white). ^*^ *p* < 0.05 and ^**^ *p* < 0.01.

We first performed immunohistochemistry (IHC) to assess HER2 expression in 48 patients with NMIBC from the Sun Yat‐Sen Memorial Hospital (SYSMH) cohort. As shown in Table S1 (Supporting Information), the expression levels of HER2 (0, 1+, 2+, and 3+) were 12.5%, 16.7%, 56.2%, and 14.6% in NMIBC, respectively. Representative images of the HER2 IHC score are shown in **Figure** **1**a. By analyzing HER2 expression and clinicopathological features, we found that HER2 had higher expression in T1 stage, high‐grade and recurrent tumor tissues than in Ta stage, low‐grade and initial tumor tissues (*p* < 0.01, *p* < 0.01, and *p* < 0.05, respectively, Figure 1b). To detect HER2 expression in cell lines, we performed qPCR and Western blotting in two breast cancer cell lines (SK‐BR‐3, [HER2 (3+)] and MDA‐MB‐231 [HER2 (‐)]), five BCa cell lines (TCCSUP, RT4, 5637, UM‐UC‐3, and T24), and an immortalized human bladder urothelium cell line (SV‐HUC‐1). HER2 was strongly expressed in SK‐BR‐3 cells but not in MDA‐MB‐231 cells. There was a low expression of HER2 in SV‐HUC‐1, UM‐UC‐3, and TCCSUP cells. The highest expression of HER2 was shown in 5637 cells and a moderate expression level in T24 and RT4 cells (Figure 1c,d). Furthermore, flow cytometry (FCM) surface staining and immunofluorescence (IF) staining were conducted to further confirm that 5637, T24, and UM‐UC‐3 represented high, moderate, and low HER2 expression levels in BCa cell lines, respectively (Figure 1e,f). Taken together, these data show that HER2 is highly expressed in some BCa cell lines and NMIBC tumor tissues and is associated with advanced pathological features and recurrence.

### 2.2 The Antitumor Effect of RC48‐ADC is Positively Associated with HER2 Expression Levels in BCa Cell Lines and Organoid Models

**Figure 2:**
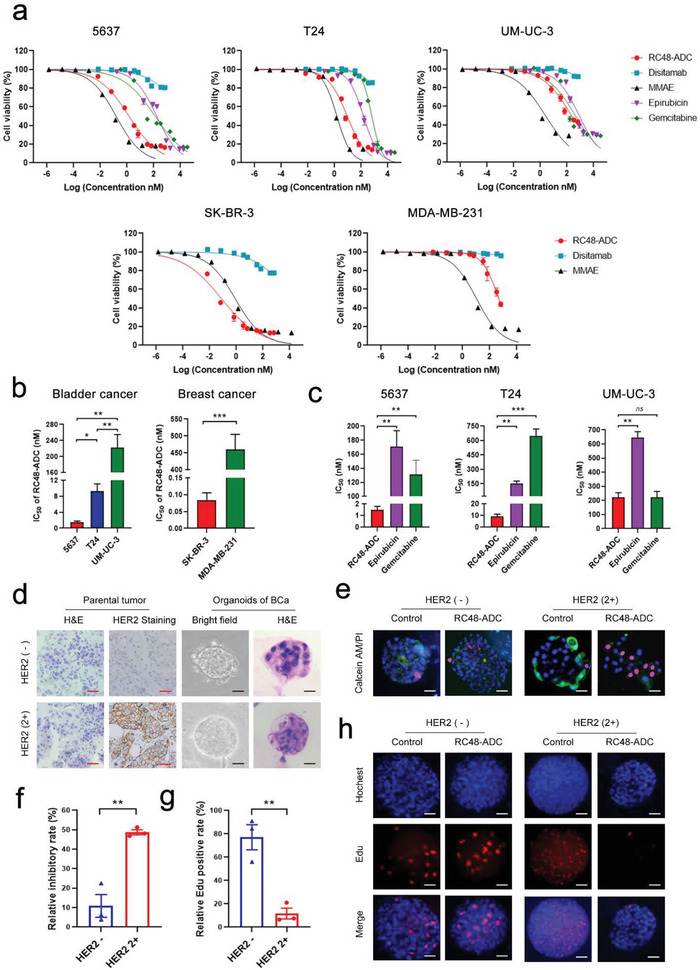
The antitumor efficacy of RC48‐ADC is positively associated with HER2 expression level in vitro. a) Effect of RC48‐ADC on the viability of BCa cell lines with different HER2 expression levels in comparison with disitamab, MMAE, and two intravesical chemotherapy agents. SK‐BR‐3 [HER2 high] and MDA‐MB‐231 [HER2 low] cells were chosen as references. b) IC_50_ of RC48‐ADC in BCa and breast cancer cell lines with different HER2 expression levels. c) IC_50_ of RC48‐ADC in BCa cell lines compared with epirubicin and gemcitabine. d) Establishment of BCa organoid models with different HER2 expression levels. Scale bars: 50 µm (red), 20 µm (black). e,f) Representative images (e) and quantification (f) of calcein‐AM/PI assays in BCa organoid models after 72 h of continuous exposure to 40 nM RC48‐ADC. Scale bars: 50 µm (white). h) Representative images and (g) quantification of EdU assays in BCa organoid models after 72 h of continuous exposure to 40 nM RC48‐ADC. Scale bars: 50 µm (white). ^*^ *p* < 0.05, ^**^ *p* < 0.01, ^***^ *p* < 0.001, and ns means not significant.

We compared the effects of RC48‐ADC on three BCa cell lines with different HER2 expression levels (5637, T24, and UM‐UC‐3) in vitro. The breast cancer cell lines SK‐BR‐3 and MDA‐MB‐231 were used as high and low HER2 expression controls, respectively. The growth inhibitory ability of RC48‐ADC, disitamab (naked monoclonal antibody (mAb) of RC48‐ADC), MMAE (cytotoxic agent conjugated with RC48‐ADC), and the intravesical chemotherapy agents commonly used in clinical practice (epirubicin and gemcitabine) were assessed. Our data showed that cells with high HER2 expression (5637, T24, and SK‐BR‐3) were more sensitive to RC48‐ADC than low HER2 expression cells (UM‐UC‐3 and MDA‐MB‐231). Disitamab showed a very weak inhibitory effect on high HER2 expression cells and no inhibitory effect on low HER2 expression cells. MMAE, a common cytotoxic drug, exhibited strong antitumor activity against both high and low HER2 expression BCa cell lines (**Figure** **2**a). Consistent with the above finding, the potency (IC_50_) and efficacy (maximum effect on cell viability) results showed that the antitumor efficacy of RC48‐ADC in cancer cell lines was positively correlated with HER2 expression level (Figure 2b; Table S1, Supporting Information).

To compare the potency and efficacy of RC48‐ADC with those of current intravesical chemotherapy agents, three BCa cell lines (5637, T24, and UM‐UC‐3) were treated with epirubicin and gemcitabine for 72 h, in conditions similar to that of RC48‐ADC. As shown in Figure 2a,c and Table S1 (Supporting Information), the potency of RC48‐ADC was 115 and 88 times higher than that of epirubicin and gemcitabine in 5637 cells (*p* < 0.01 and *p* < 0.01, respectively) and 17 and 70 times higher in T24 cells (*p* < 0.01 and *p* < 0.001, respectively). However, RC48‐ADC showed threefold higher potency than epirubicin (*p* < 0.01) and was equal to gemcitabine (*p* > 0.05) in UM‐UC‐3 cells. These data indicate that RC48‐ADC showed better antitumor efficacy than current intravesical chemotherapy agents in vitro, especially in high HER2 expression BCa cells.

BCa organoid models were then established to further evaluate the effect of RC48‐ADC with different HER2 expression levels (Figure 2d). We found that the cell death rate of the high HER2 expression BCa organoids was significantly higher than that of the low HER2 expression organoids (48.77% vs 10.89%; *p* < 0.01) (Figure 2e,f). Furthermore, the ethynyl deoxyuridine (EdU) cell proliferation assay revealed that RC48‐ADC exerted more powerful inhibitory effects on high HER2 expression BCa organoids than on low HER2 expression organoids (11.71% vs 77.21%; *p* < 0.01) (Figure 2g,h). Collectively, these results demonstrated that RC48‐ADC exerts promising antitumor activity in vitro and this effect is dependent on HER2 expression.

### 2.3 RC48‐ADC Inhibits BCa Cells by Inducing G2/M Cell Cycle Arrest and Cell Apoptosis

**Figure 3:**
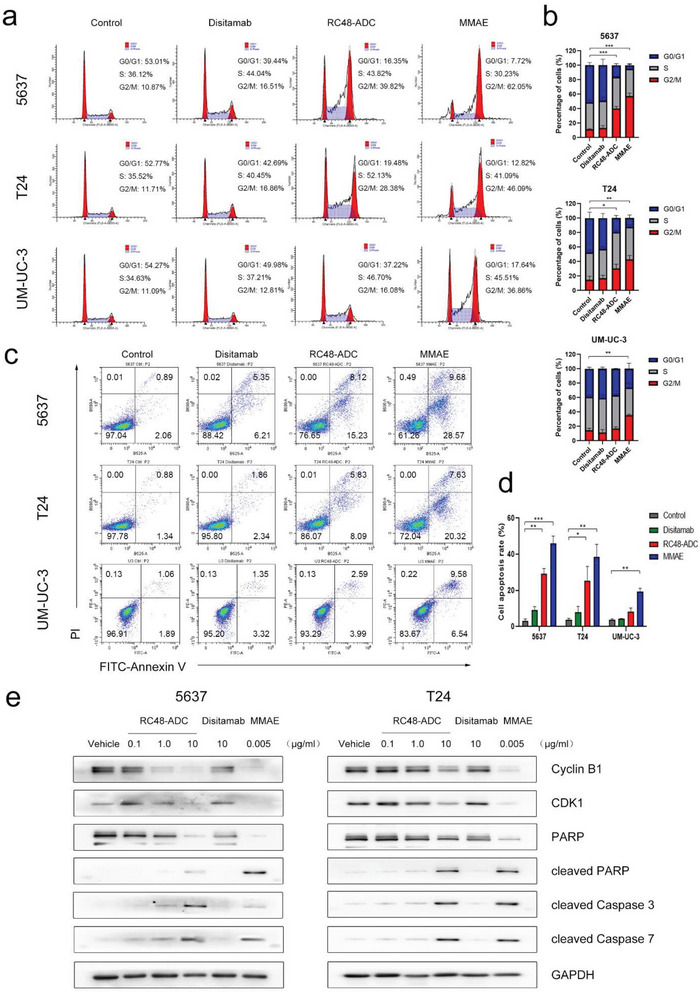
RC48‐ADC inhibits BCa cells by inducing G2/M cell cycle arrest and cell apoptosis. a) Representative images and (b) quantification of the cell cycle in three BCa cell lines treated with RC48‐ADC, disitamab, and MMAE at an equivalent molar concentration (6.7 nM) for 48 h. c) Representative images and (d) quantification of cell apoptosis in three BCa cell lines treated with RC48‐ADC, disitamab, and MMAE at an equivalent molar concentration (6.7 nM) for 48 h. e) The expression of RC48‐ADC target proteins related to G2/M cell cycle arrest and caspase‐dependent cell apoptosis detected by Western blotting in 5637 and T24 cells. Cells were treated with RC48‐ADC (0.1, 1.0, 10 µg mL^−1^), disitamab (10 µg mL^−1^), and free MMAE (0.005 µg mL^−1^) for 48 h before being harvested and lysed. ^*^ *p* < 0.05, ^**^ *p* < 0.01, and ^***^ *p* < 0.001.

To evaluate the antitumor roles of RC48‐ADC in BCa cells, we detected cell cycle and apoptosis by FCM and compared them with those cells treated with MMAE and disitamab. MMAE is a tubulin inhibitor that disrupts cell division by inhibiting microtubule assembly, thus interrupting cell division in the G2/M phase.^[^ ^23^ ^]^ Consistent with previous studies, we found that most 5637, T24, and UM‐UC‐3 cells treated with MMAE were blocked in the G2/M phase (*p* < 0.001, *p* < 0.01, and *p* < 0.01, respectively). RC48‐ADC could also significantly arrest 5637 and T24 cells with high expression of HER2 in the G2/M phase (*p* < 0.001 and *p* < 0.05, respectively), but for UM‐UC‐3 cells with low expression of HER2, no obvious blocking effect was observed after RC48‐ADC treatment at the same concentration (*p* > 0.05). For disitamab, no significant cycle arrest effect was observed in these three BCa cell lines with different HER2 expression levels (**Figure** **3**a,b). The apoptosis analysis revealed the same trend as the cell cycle arrest analysis. MMAE induced the highest apoptosis rate in 5637, T24, and UM‐UC‐3 cells (*p* < 0.001, *p* < 0.01, and *p* < 0.01, respectively). RC48‐ADC induced more cell apoptosis in 5637 and T24 cells (*p* < 0.01 and *p* < 0.05, respectively) with high expression of HER2 than in UM‐UC‐3 (*p* > 0.05) cells with low expression of HER2. No obvious apoptotic cells were observed after treatment with disitamab in 5637, T24, and UM‐UC‐3 cells (Figure 3c,d).

To investigate the mechanism of RC48‐ADC in BCa cells, we detected some cell cycle‐ and apoptosis‐related proteins by Western blotting (Figure 3e). The data showed that the G2/M phase‐related proteins cyclin dependent kinase 1 (CDK1) and cyclin B1 were significantly downregulated in 5637 and T24 cells after exposure to RC48‐ADC in a dose‐dependent manner. A similar result was observed in the MMAE (0.005 µg mL^−1^) group but not in the disitamab (10 µg mL^−1^) group. Next, we examined the apoptosis‐related proteins caspase 3, caspase 7, and poly adenosine‐diphosphate (ADP) ‐ribose polymerase (PARP). Cleaved caspase 3, cleaved caspase 7, and cleaved PARP were expressed in 5637 and T24 cells after exposure to both RC48‐ADC and MMAE but not after exposure to disitamab. Overall, these results suggested that RC48‐ADC could arrest the cell cycle in the G2/M phase by downregulating CDK1 and cyclin B1 and inducing caspase‐dependent cell apoptosis in high HER2 expression BCa cells.

### 2.4 Intravesical RC48‐ADC Shows Promising Antitumor Activity in an Orthotopic BCa Mouse Model

**Figure 4:**
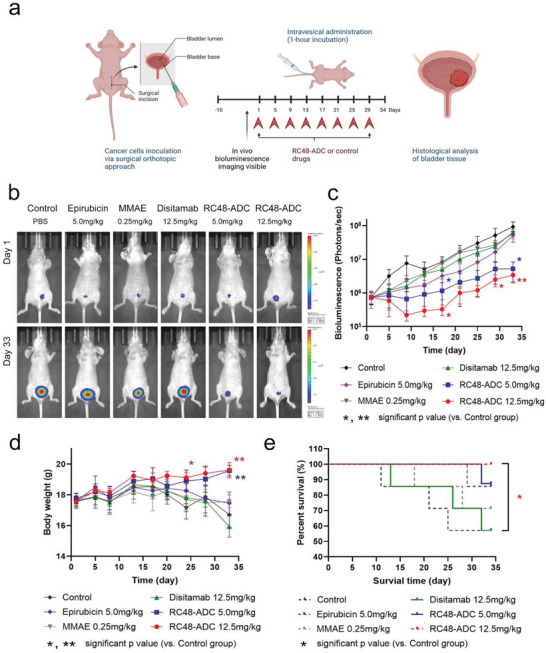
Intravesical RC48‐ADC shows promising antitumor activity in an orthotopic BCa mouse model. a) Schematic illustration of intravesical instillation treatment in the orthotopic BCa model. An orthotopic BCa model with luciferase‐expressing T24 cells was established and tumor growth was monitored by bioluminescent imaging. Treatment was started on day 10 after xenograft model construction and a one‐month intravesical instillation schedule was carried out; agents were administered at the indicated concentrations every 4 days. The tumor size and body weight of the mice were monitored during treatment. At the end of the experiment, the bladders of the mice were dissected and removed for weighing, fixation, sectioning, and hematoxylin‐eosin (HE) staining to evaluate tumor status. b) Representative images of bioluminescence (orthotopic‐xenograft bladder tumor) in each group on the first and last day of monitoring. c) In vivo tumor inhibition effect monitored by bioluminescence. d) Average body weights of mice in different groups during intravesical treatments. e) Cumulative survival rates of mice in different groups. A log‐rank test was performed to compare survival among groups.

To evaluate the efficacy of RC48‐ADC intravesical treatment in vivo, we first established an orthotopic BCa model with luciferase‐expressing T24 cells and monitored tumor growth by bioluminescent imaging. 44 orthotopic BCa model mice were grouped and treated with RC48‐ADC (eight mice in each group), disitamab (seven mice), MMAE (seven mice), epirubicin (seven mice), and PBS (seven mice) (**Figure** **4**a). The treatment with intravesical instillation of RC48‐ADC at doses of 5.0 and 12.5 mg kg^−1^ every 4 days showed more significant antitumor effects than other treatments (*p* < 0.05 and *p* < 0.01, respectively, Figure 4b,c). Tumor growth inhibition by RC48‐ADC was observed 2 weeks after the first treatment, and treatment with RC48‐ADC at a dose of 12.5 mg kg^−1^ showed more sustained antitumor effects than treatment with 5.0 mg kg^−1^. In contrast, 12.5 mg kg^−1^ disitamab, 0.25 mg kg^−1^ MMAE, and 5.0 mg kg^−1^ epirubicin at the same frequency showed no or little efficacy. The body weight of mice was stable in both groups of RC48‐ADC at doses of 5.0 and 12.5 mg kg^−1^ but decreased in other treatment groups (*p* < 0.01 and *p* < 0.01, respectively, Figure 4d). Furthermore, the higher dose of RC48‐ADC significantly prolonged the survival of mice compared with that in the PBS and disitamab groups (*p* < 0.05, Figure 4e).

**Figure 5:**
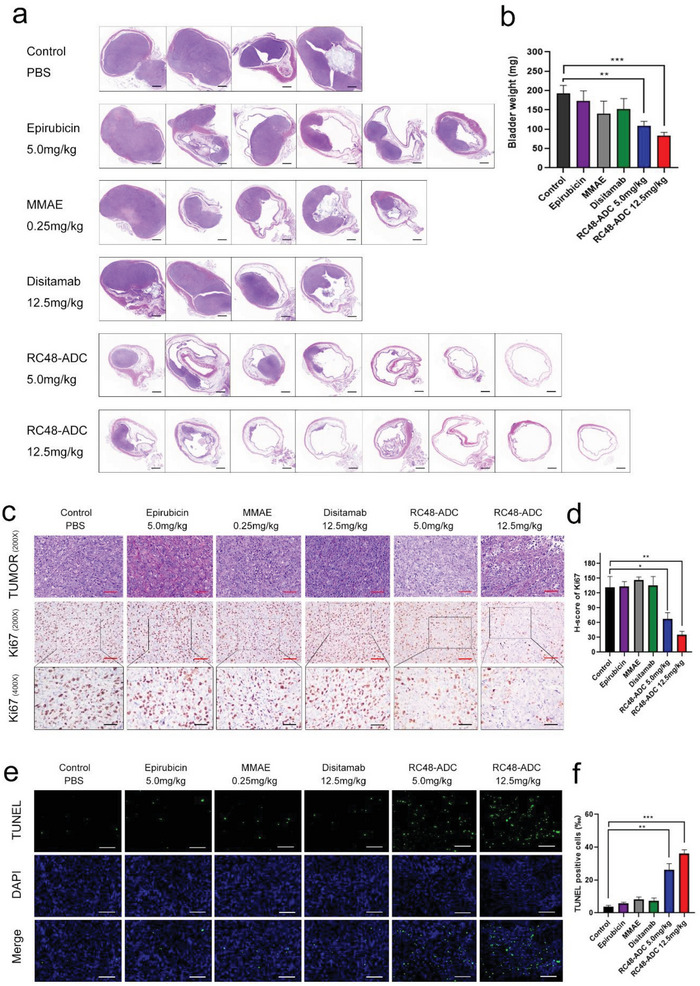
Intravesical RC48‐ADC suppresses tumor growth in vivo. a) HE‐stained slices of bladders and (b) weight measurements of the collected bladder at the end of the in vivo study. Scale bars: 1 mm (black). c) Representative images of Ki67 expression in the tumor examined by IHC. Scale bars: 100 µm (red), 50 µm (black). d) Quantification of the H‐score of Ki67 IHC in each group. e) Representative images of tumor apoptosis detected by TUNEL assay. Scale bars: 100 µm (white). f) Quantification of TUNEL‐positive cell proportions in each group. ^*^ *p*< 0.05, ^**^ *p* < 0.01, and ^***^ *p* < 0.001.

Bladders were removed from the mice that survived until the end of the study for weighing, fixation, sectioning, and hematoxylin‐eosin (HE) staining to determine the tumor status. First, we found that most of the bladders from the RC48‐ADC‐treated groups were free of tumors or had only small tumors when visualized with the naked eye. However, large tumors inside the bladder were observed in the disitamab, MMAE, and epirubicin treatment groups and the control group (Figure S1, Supporting Information). Furthermore, HE staining confirmed that tumors were present in 50.0% (4/8) of the mice receiving 12.5 mg kg^−1^ RC48‐ADC, 71.4% (5/7) of the mice receiving 5.0 mg kg^−1^ RC48‐ADC, and 100% of the mice receiving disitamab (4/4), MMAE (5/5), and epirubicin (6/6). The tumors in the RC48‐ADC treatment group were significantly smaller than those in the other treatment groups and the control group. Moreover, RC48‐ADC inhibited tumor growth more effectively at a dose of 12.5 mg kg^−1^ than at 5.0 mg kg^−1^ (**Figure** **5**a). The bladder weights of groups treated with RC48‐ADC at dose of 5.0 and 12.5 mg kg^−1^ were significantly less than those of the other treatment groups and the control group (*p* < 0.01 and *p* < 0.001, respectively, Figure 5b). Consistent with the in vitro results, IHC staining of the proliferation marker Ki67 and erminal deoxynucleotidyl transferase‐mediated dUTP nick end labelling (TUNEL) assays revealed that tumors derived from groups treated with RC48‐ADC at dose of 5.0 and 12.5 mg kg^−1^ had lower proliferation activity (*p* < 0.05 and *p* < 0.01, respectively, Figure 5c,d) and a higher apoptotic rate than other treatment groups or the control group (*p* < 0.01 and *p* < 0.001, respectively, Figure 5e,f). Collectively, these data strongly indicated that intravesical RC48‐ADC shows satisfactory antitumor activity in the orthotopic mouse BCa model.

### 2.5 Intravesical RC48‐ADC Exerted Tolerable Safety in a Repeated‐Dose Toxicity Study

**Figure 6:**
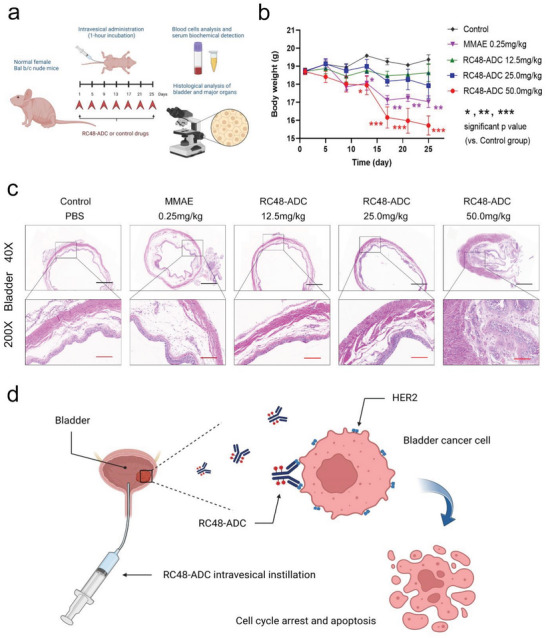
Intravesical RC48‐ADC showed tolerable safety in vivo. a) Schematic illustration of intravesical RC48‐ADC in a repeated‐dose toxicity study. Three increasing dose groups of RC48‐ADC were used for the repeated‐dose toxicity experiment. A free MMAE group and PBS blank control group were used as controls. Each group included six nude mice, and the body weight of the mice was monitored during the experiment. At the end of the experiment, all mice were sacrificed, and whole blood and plasma were collected for blood cell and serum biochemical analysis. The bladder and major organs, including the kidney, liver, spleen, heart, and lung, were removed for fixation, sectioning and HE staining to determine local and systemic adverse reactions. b) Average body weights of mice in different groups after intravesical treatments. c) HE‐stained slices of bladders at the end of the repeated‐dose toxicity study. Scale bars: 1 mm (black), 200 µm (red). d) Schematic diagram depicting BCa cell apoptosis induced by intravesical instillation of RC48‐ADC in a HER2 expression‐dependent manner.

To evaluate the potential toxicity of intravesical RC48‐ADC, intravesical instillation of RC48‐ADC or control was administered every four days to female BALB/c nude mice until visible adverse effects occurred (**Figure** **6**a). Three RC48‐ADC dose groups were set in the repeated‐dose toxicity experiment. The first dose was 12.5 mg kg^−1^, which was proven to have a satisfactory antitumor effect in an orthotopic mouse BCa model. The second and third doses were 25.0 and 50.0 mg kg^−1^. We also set a control group of 0.25 mg kg^−1^ MMAE, which contained the same amount of MMAE as 12.5 mg kg^−1^ RC48‐ADC. In addition, a PBS blank control group was set up. Each group included six nude mice.

By observing and recording the body weight of the nude mice, we found a significant decrease in body weight in the 50.0 mg kg^−1^ RC48‐ADC group and the 0.25 g kg^−1^ MMAE group compared with the PBS control group from the 4th treatment (*p* < 0.001 and *p* < 0.01, respectively, Figure 6b). The repeated dose treatment was stopped at the 7th treatment when two mice in the 50.0 mg kg^−1^ RC48‐ADC group died. All mice were sacrificed, and whole blood and plasma were collected for blood cell and serum biochemical analysis. As shown in Table S2 (Supporting Information), blood cell analysis showed that there were no abnormalities in various cells, including red blood cells, white blood cells, and platelets, in each RC48‐ADC dose group, the 0.25 mg kg^−1^ MMAE group, or the PBS control group. However, serum biochemical analysis revealed mild renal function impairment in the 50.0 mg kg^−1^ RC48‐ADC dose group. No abnormalities were found in liver function or myocardial injury indexes.

Furthermore, the bladder and major organs, including the kidney, liver, spleen, heart, and lung, were removed from all the nude mice for fixation, sectioning, and HE staining to determine local and systemic adverse effects. HE staining revealed that for three mice in the 50.0 mg kg^−1^ RC48‐ADC group, the bladder wall was significantly thickened and the volume of the bladder was reduced. The bladder submucosa showed more fibre proliferation, vascular dilatation, and more inflammatory cell infiltration. No obvious bladder inflammation was observed in the 12.5 and 25 mg kg^−1^ treatment groups (Figure 6c). HE staining of major organs showed that there were no significant organic changes in the kidney, liver, spleen, heart, and lung of mice in each group (Figure S2, Supporting Information). Collectively, these data demonstrate that administration of RC48‐ADC by intravesical instillation is safe within the range of effective therapeutic doses. However, the risk of bladder inflammation and renal function damage could increase at a high dose.

## 3 Discussion

NMIBC is typically treated with TURBT, with the addition of intravesical therapy for patients with a high risk of recurrence. BCG is recommended for high‐risk NMIBC. However, treatment options are limited for BCG‐intolerant or BCG‐unresponsive disease. At present, radical cystectomy (RC) remains the standard of care for high‐risk NMIBC patients who have failed BCG treatment.^[^ ^5^ ^]^ Therefore, it is particularly important to explore new intravesical agents for bladder‐preserving therapy. RC48‐ADC is well tolerated and shows a promising objective response rate (ORR) in HER2‐positive advanced BCa.^[^ ^11^ ^]^ However, the applicability of RC48‐ADC for intravesical therapy of NMIBC remains unclear. This study demonstrated the satisfactory antitumor effects and safety of intravesical administration of RC48‐ADC through multiple preclinical models (Figure 6d). These findings provide evidence to support further investigation of intravesical instillation of RC48‐ADC in patients with NMIBC.

Although radical cystectomy (RC) can achieve the best oncological control for patients, the possibility of overtreatment and the decline in quality of life caused by whole bladder resection makes it difficult for many patients to accept it. For patients who refuse or are not suitable for RC, current bladder‐preserving therapies supported by clinical trials include immunotherapy (pembrolizumab), gene therapy (nadofaragene firadenovec), intravesical combination therapy (gemcitabine/docetaxel), device‐delivered therapy (photodynamic therapy or bladder intracavitary hyperthermic chemotherapy) and so on. The results from phase II‐III clinical studies have shown that these new therapies have efficacy rates (3 and 12 months) of 40–82% and 17–44%, respectively, and the long‐term efficacy remains to be further observed^[^ ^24^, ^25^, ^26^, ^27^ ^]^ Recently, targeted agents such as the fibroblast growth factor receptor (FGFR) inhibitor erdafitinib and ADCs have demonstrated robust efficacy after progression on prior chemotherapy and immunotherapy in advanced BCa.^[^ ^28^ ^]^ Compared to erdafitinib, ADCs are available to more BCa patients due to the high expression rate of target proteins. A head‐to‐head comparison of expression differences in HER2, NECTIN4, and TROP2 demonstrated that the majority of BCa expressed at least one ADC target.^[^ ^29^ ^]^ In this study, we revealed that HER2 was also upregulated in NMIBC, and the overexpression rate of HER2 (2+ or 3+) was 70.8%. Furthermore, HER2 showed higher expression in the T1 stage, high‐grade tumor tissues, and recurrent disease. These results were consistent with those of a previous study that indicated that HER2 overexpression was a risk factor for recurrence and progression in NMIBC.^[^ ^20^ ^]^ Hence, based on the promising efficacy of systemic administration and the expression of HER2 in NMIBC, we speculated that the application of RC48‐ADC for intravesical therapy in NMIBC may be effective.

Our study revealed that BCa cell lines with higher HER2 expression were more sensitive to RC48‐ADC and had lower IC_50_ values. Furthermore, we established organoid models of NMIBC and found that the inhibition of proliferation and induction of apoptosis by RC48‐ADC were significantly better in high HER2 expression organoids than in those with low HER2 expression. This is the first time that the antitumor activity of RC48‐ADC against NMIBC was confirmed using organoid models. In vivo, we performed intravesical instillation with different agents in an orthotopic BCa mouse model and found that RC48‐ADC could significantly inhibit tumor growth in the 5.0 and 12.5 mg kg^−1^ groups, while disitamab, MMAE, and even epirubicin had no significant inhibitory effect on BCa with a relatively high tumor burden. The results demonstrate that intravesical administration of RC48‐ADC can effectively inhibit tumor growth, even in those with a high tumor burden. Considering that the intravesical agents were administered after surgical removal of visible tumors in clinical practice, a situation in which the patient presents a much lower tumor burden, it is presumed that the application of RC48‐ADC could kill microscopic residual tumors and prolong time to tumor recurrence.

After establishing that intravesical administration of RC48‐ADC was effective in inhibiting BCa growth, its safety profile was explored. We conducted repeated toxicity experiments at three increasing doses and evaluated the potential toxicity of RC48‐ADC intravesical administration. There were no detectable local or systemic toxicities at the therapeutic dose. However, local inflammation of the bladder and renal impairment were detected with an intravesical dosage of 50.0 mg kg, suggesting the toxicity dose of RC48‐ADC. Based on the conversion, the dose in these three mice corresponded to a human dose of 240 mg, suggesting that patients may suffer from TRAEs with intravesical administration of 240 mg RC48‐ADC, which provides a basis for the selection of drug dosage in subsequent human clinical trials.

Recently, enfortumab vedotin (EV), a Nectin4‐directed ADC, was evaluated in a phase I clinical trial (NCT05014139) for intravesical administration in the treatment of NMIBC. Publicly available data are limited, but preclinical models have demonstrated its antitumor activity in human BCa cells expressing Nectin‐4. Intravesical administration of enfortumab vedotin (EV) was well tolerated with no detectable local or systemic toxicities with low and transient systemic absorption.^[^ ^30^ ^]^ In addition, the VISTA study (NCT02449239) is a phase III study evaluating another ADC, vicinium, which targets epithelial cell adhesion molecule (EpCAM), in high‐risk BCG‐unresponsive NMIBC patients. The study showed that the complete response rate (CRR) of carcinoma in situ (CIS) was 40%, and the recurrence‐free rate of papillary carcinoma was 71% after 3 months of treatment. At 12 months, 17% of CIS patients still maintained complete response (CR) status without progressive or metastatic disease. Furthermore, intravesical vicinium was proven to be safe and well tolerated, with a grade 3 to 5 adverse events rate of 4%.^[^ ^31^ ^]^ These two ongoing trials revealed the feasibility of ADCs for intravesical instillation. However, considering that there is still no specific therapeutic target for BCa, further verification of other targeted drugs is necessary to promote the clinical application of ADC intravesical therapy. HER2 is recognized as a potential candidate for targeted antibody therapy. RC48‐ADC, as a HER2‐targeting ADC, shows promising efficacy in HER2‐positive advanced BCa.^[^ ^11^, ^21^, ^22^ ^]^ In this study, RC48‐ADC is also proved to be effective and safe with intravesical instillation in preclinical models of BCa. Collectively, previous studies and our study have demonstrated the unique advantages of ADCs in terms of the efficacy and safety of intravesical administration for the treatment of NMIBC.

The limitation of this study is that the pretreatment tumor burden was relatively high in the orthotopic BCa model, which is inconsistent with the low tumor burden seen after surgical resection in clinical practice. However, even in this case, intravesical instillation with RC48‐ADC was effective and was more efficacious than chemotherapeutic agents, which suggests that ADCs represented by RC48‐ADC may have good prospects in preventing NMIBC recurrence if administered by intravesical instillation. Based on this preclinical study, a phase I/II clinical trial with intravesical instillation of RC48‐ADC for the treatment of HER2‐positive high‐risk NMIBC is underway. In the clinical trial, we will explore the efficacy and safety of intravesical RC48‐ADC treatment in high‐risk NMIBC patients, including those who have failed or refused BCG treatment or were not eligible for BCG treatment. By conducting further clinical studies, we can better clarify the safety, effectiveness, and pharmacokinetics of intravesical RC48‐ADC in the treatment of high‐risk NMIBC. Intravesical delivery of RC48‐ADC may make it possible to preserve the bladder for patients with HER2‐positive high‐risk NMIBC and may provide a new strategy for the individualized treatment of high‐risk NMIBC.

## 4 Conclusion

Administration of RC48‐ADC demonstrated promising antitumor efficacy in human BCa cells expressing HER2 in vitro and in vivo. Intravesical administration of RC48‐ADC was well tolerated with no detectable local or systemic toxicities at the therapeutic dose. These preclinical findings provide valuable evidence to support further clinical trials of intravesical RC48‐ADC in patients with NMIBC to improve therapeutic outcomes.

## 5 Experimental Section

### Clinical Samples

Forty‐eight snap‐frozen, formalin‐fixed, paraffin‐embedded tissue samples were obtained from patients who underwent surgery at Sun Yat‐Sen Memorial Hospital (SYSMH) of Sun Yat‐Sen University. The study was approved by the Committees for Ethical Review of Research Involving Human Subjects of Sun Yat‐Sen University (2022‐KY‐060). Patient demographics and clinical characteristics are summarized in Table S3 (Supporting Information).

### Immunohistochemistry (IHC) Staining and Scoring Analysis

The IHC experiment and IHC score calculations were conducted as previously described.^[^ ^32^ ^]^ The primary antibody used was the Ventana anti‐HER2/Neu (4B5) rabbit monoclonal antibody (mAb), and staining was performed with the Ultra‐View Universal DAB Detection Kit (Roche, Switzerland). IHC scores were reviewed by two independent pathologists at the central laboratory according to the HER2 test guidelines for cancer.^[^ ^33^ ^]^ Images were obtained by an ECLIPSE Ti microscope system (Nikon, Japan).

### Cell Lines and Cell Culture

Human BCa cell lines (T24, UM‐UC‐3, 5637, RT4, and TCCSUP), human bladder epithelial cells (SV‐HUC‐1), and human breast cancer cell lines (SK‐BR‐3 and MDA‐MB‐231) were purchased from the American Type Culture Collection (ATCC, USA). RPMI 1640 containing 10% foetal bovine serum (FBS) was used to culture T24, 5637, and RT4 cells. F‐12K medium containing 10% FBS was used to culture SV‐HUC‐1 cells. Other cells, including UM‐UC‐3, TCCSUP, SK‐BR‐3, and MDA‐MB‐231 cells, were cultured in dulbecco's modified eagle medium (DMEM) supplemented with 10% FBS. All cell lines were cultured in a humidified incubator with 5% CO_2_ at 37 °C.

### BCa Organoid Culture

Fresh BCa tissues were acquired from patients after obtaining informed consent and placed in tissue storage solution (130‐100‐008, Miltenyi, Germany) for transportation. The clinical information of these patients is described in Table S4 (Supporting Information). Six fresh NMIBC tumor samples were washed three times in Phosphate‐buffered saline (PBS) containing 50 µg mL^−1^ nystatin, 500 µg mL^−1^ streptomycin and 500 U penicillin, and the samples were then cut into small particles and digested in Adv DMEM/F‐12 (12634028, Thermo Fisher, USA) containing 5 mg mL^−1^ collagenase type II (LS004176, Worthington, USA), 10 µg mL^−1^ DNAse I (LS002138, Worthington, USA) and 10 µM ROCK inhibitor (Y‐27632, Sigma, USA) at 37 °C for 30 min. To remove undigested tissue, a 100 µm cell strainer was used, and the suspension was centrifuged to pellet the cells. The collected cells were resuspended in complete culture media (specific ingredients are provided in Table S5, Supporting Information) containing a Matrigel matrix at a ratio of 1:2. Cells in droplets (10,000 cells/30 µl) were plated in a 48‐well plate. IHC confirmed that three patients were HER2 2+, and three were HER2 negative.

### Ribonucleic Acid (RNA) Isolation, Quantitative Reverse Transcription Polymerase Chain Reaction (qRT‐PCR) and Western Blotting

Total RNA was extracted from cells using TRIzol (TaKaRa, Japan) according to the manufacturer's instructions and used as a template for reverse transcription using the PrimeScript RT–PCR kit (TaKaRa, Japan). qRT–PCR was performed on a LightCycler 480 system with a SYBR Green PCR kit (TaKaRa, Japan) as previously described.^[^ ^34^, ^35^ ^]^ Gene expression was calculated using the 2‐△Ct method. All the primers used in this study are listed in Table S6 (Supporting Information).

Western blotting was performed as previously described.^[^ ^36^ ^]^ The primary antibodies used in this study are listed in Table S7 (Supporting Information). Glyceraldehyde‐3‐phosphate dehydrogenase (GAPDH) was used as a loading control. The polyvinylidenefluoride (PVDF) membranes were incubated with secondary antibodies (anti‐mouse or anti‐rabbit, Proteintech, China) and visualized using Immobilon‐enhanced chemiluminescence (Millipore, USA).

### Immunofluorescence (IF) and Cell Surface Flow Cytometry (FCM) Staining

IF was performed as described previously.^[^ ^37^ ^]^ Cells were fixed in confocal culture dishes with 4% paraformaldehyde and incubated with HER/ErbB2 antibody (anti‐rabbit, Proteintech, China) at 4 °C overnight. Then, the cells were further incubated with an anti‐rabbit secondary antibody (Alexa Fluor‐488, goat anti‐rabbit, Immunoway, USA), followed by 4′,6‐diamidino‐2′‐phenylindole (DAPI) to stain nuclei. A Leica TCS SP8 confocal microscope system (Leica Microsystems, Germany) was used to observe and acquire images.

HER2 expression on the surface of cells was determined by FCM using fluorescein isothiocyanate (FITC)‐conjugated anti‐human ErbB2/HER2 antibody (Biolegend, Fell, Germany). FCM analysis was performed with CytoFLEX flow cytometers (Beckman Colter, USA), and data were analyzed using FlowJo Version 10.0.0 (FlowJo, Ashland, OR) software.

### In Vitro Cytotoxicity Assay

Cells were seeded in 96‐well culture plates. The cytotoxicity of RC48‐ADC and disitamab were evaluated at concentrations of 0.001, 0.01, 0.1, 0.5, 1, 5, 10, 50, and 100 µg mL^−1^ in BCa 5637, T24, UM‐UC‐3, and breast cancer SK‐BR‐3 and MDA‐MB‐231 cells in vitro. The effect of free MMAE was measured at concentrations of 0.000001, 0.00001, 0.0001, 0.001, 0.01, 0.1, 1, 10, and 100 ng mL^−1^ as a control. The effects of epirubicin and gemcitabine were assayed at concentrations of 0.1, 1.0, 10, 50, 100, 500, 1000, 5000, and 10000 ng mL^−1^ in BCa cells. All concentrations were converted to nM for comparison between drugs. The cells were cultured with these agents for 72 h, then cell viability was detected using the Cell Counting Kit‐8 (CCK8) assay and half‐inhibitory concentration (IC_50_) was computed to evaluate the antitumor potency of drugs.

Calcein acetoxymethyl ester/propidium iodide (calcein‐AM/PI) co‐staining experiments were conducted to determine the live/dead cell fraction in the BCa organoid models as previously described.^[^ ^38^ ^]^ The samples were observed under a fluorescence microscope (Nikon, ECLIPSE Ni‐U, Japan).

### Cell Cycle and Apoptosis Analysis

The effects of RC48‐ADC (1 µg mL^−1^) on the cell cycle were examined using BCa cell lines (5637, T24, and UM‐UC‐3). The equivalent molar concentrations (6.7 nm) of disitamab and MMAE were used. Cells were seeded in 6‐well culture plates and then cultured for 24 h before adding the different drugs. The Cell Cycle Analysis Kit (4A Biotech, China) was used for PI labeling, and the Apoptosis Analysis Kit (4A Biotech, China) was used for FITC and PI staining after incubating the cells at 37 °C for 48 h. These procedures were performed as described previously.^[^ ^39^ ^]^ The results were analyzed by CytoFLEX flow cytometers (Beckman Coulter, USA).

An ethynyl deoxyuridine (EdU) assay was performed to detect the proportion of cells in an S phase. A terminal deoxynucleotidyl transferase‐mediated dUTP nick end labeling (TUNEL) assay was conducted to detect the proportion of apoptotic cells using the In Situ Cell Death Detection Kit (Roche, Switzerland) following the manufacturer's instructions and as described previously.^[^ ^40^ ^]^

### Orthotopic Bladder Tumor Mouse Model

All animal studies were conducted with the approval of the Sun Yat‐Sen University Institutional Animal Care and Use Committee. Eight‐week‐old female BALB/c nude mice were purchased from Bestest Biotechnology Co., Ltd. (ZhuHai, China) and housed in SPF barrier facilities. A previously described orthotopic bladder tumor mouse model was used in this study,^[^ ^41^ ^]^ with some modifications (Figure S3, Supporting Information). The orthotopic xenograft bladder tumor was monitored and imaged using a bioluminescence imaging system (IVIS 656 Spectrum in vivo imaging system, PerkinElmer, USA).

### Intravesical Treatment Assay

To investigate antitumor activity via intravesical treatment, the in vivo efficacy of RC48‐ADC was compared with that of the naked HER2 antibody disitamab, the free drug MMAE, and the current intravesical chemotherapy agent epirubicin. PBS was used as a blank control. All agents were administered at a volume of 50 µl via the same catheterization technique as described.^[^ ^42^ ^]^ The retention time in the bladder was ≈1 h until the mice awoke from anaesthesia and spontaneously urinated. The treatment process is shown in Figure 4a.

### Repeated‐Dose Toxicity Assay

To evaluate the potential toxicity of intravesical treatment, intravesical instillation of RC48‐ADC or control agents was performed every four days to eight‐week‐old female Balb/c nude mice until visible adverse effects occurred. The treatment process is shown in Figure 6a.

### Statistical Analysis

All experiments in the present study were performed three independent times. Quantitative data were presented as the mean ± standard deviation (SD). The differences between the two groups were analyzed using unpaired Student's t‐tests and one‐way analysis of variance (ANOVA), which was performed when more than two groups were compared. Spearman's correlation analysis was performed to determine the correlation between HER2 expression and clinicopathological variables. Cumulative survival time was calculated using the Kaplan–Meier method and analyzed by the log‐rank test. All statistical analyses were performed using SPSS 25.0 software. *p* < 0.05 was considered to indicate statistical significance.

### Ethics Approval and Consent to Participate

The study was approved by the Institutional Review Board of Sun Yat‐Sen Memorial Hospital of Sun Yat‐Sen University (2022‐KY‐060), and the requirement for informed consent was waived. The animal use protocol was approved by the Institutional Animal Care and Used Committee of Sun Yat‐Sen University (SYSU‐IACUC‐2022‐001010).

## Conflict of Interest

All authors declare no conflicts of interest.

## Author Contributions

X.W.H., X.C., and H.J.W. contributed equally to this work. Y.H.Z., T.X.L., and X.C. conceived and designed this study. X.W.H., X.C., and H.J.W. conducted the main experiment and performed data analysis. Q.C.X. and K.H.X. collected clinical samples and performed clinicopathological characteristics analysis. Y.F.Z and Z.P.C assisted in data and diagraph analyzes. Y.Q.L. analyzed clinical samples with IHC. G.Y.L. and H.L. conducted BCa organoid culture and relevant functional experiments. J.M.F. provided technical and material support. X.W.H., X.C., Y.H.Z., and T.X.L. wrote and reviewed the manuscript. All authors read and approved the final manuscript. Authorship order among the co‐first authors was determined according to their relative contribution.

## Supporting information

Supporting Information

## Data Availability

The data that support the findings of this study are available from the corresponding author upon reasonable request.
