# Supplementary material for: A HER2‐targeted Antibody‐Drug Conjugate, RC48‐ADC, Exerted Promising Antitumor Efficacy and Safety with Intravesical Instillation in Preclinical Models of Bladder Cancer
Source: Adv Sci (Weinh). 2023 Oct 12;10(32):2302377. doi: 10.1002/advs.202302377 (PMC10646285; doi:10.1002/advs.202302377)
Supplement: Supplementary file 1 — Supporting Information [file ADVS-10-2302377-s001.pdf]

ADVANCED SCIENCE
Open Access

Supporting Information

for *Adv. Sci.*, DOI 10.1002/advs.202302377

A HER2-targeted Antibody-Drug Conjugate, RC48-ADC, Exerted Promising Antitumor Efficacy and Safety with Intravesical Instillation in Preclinical Models of Bladder Cancer

Xuwei Hong, Xu Chen, Hongjin Wang, Qingchun Xu, Kanghua Xiao, Yuanfeng Zhang, Zepai Chi, Yeqing Liu, Guangyao Liu, Hong Li, Jianmin Fang, Tianxin Lin* and Yonghai Zhang**

Supplementary materials for:

A HER2-targeted antibody-drug conjugate, RC48-ADC, exerted promising antitumour efficacy and safety with intravesical instillation in preclinical models of bladder cancer

Running title: Intravesical instillation with RC48-ADC in preclinical models

Xuwei Hong, Xu Chen, Hongjin Wang, Qingchun Xu, Kanghua Xiao, Yeqing Liu, Guangyao Liu, Hong Li, Jianmin Fang, Tianxin Lin*, and Yonghai Zhang**

Table of contents

Supplementary figures……………………………………………2-4

Supplementary tables……………………………………………5-10

Supplementary Figures

RC48-ADC 12.5mg/kg

RC48-ADC 5.0mg/kg

Disitamab 12.5mg/kg

MMAE 0.25mg/kg

Epirubicin 5.0mg/kg

Control PBS

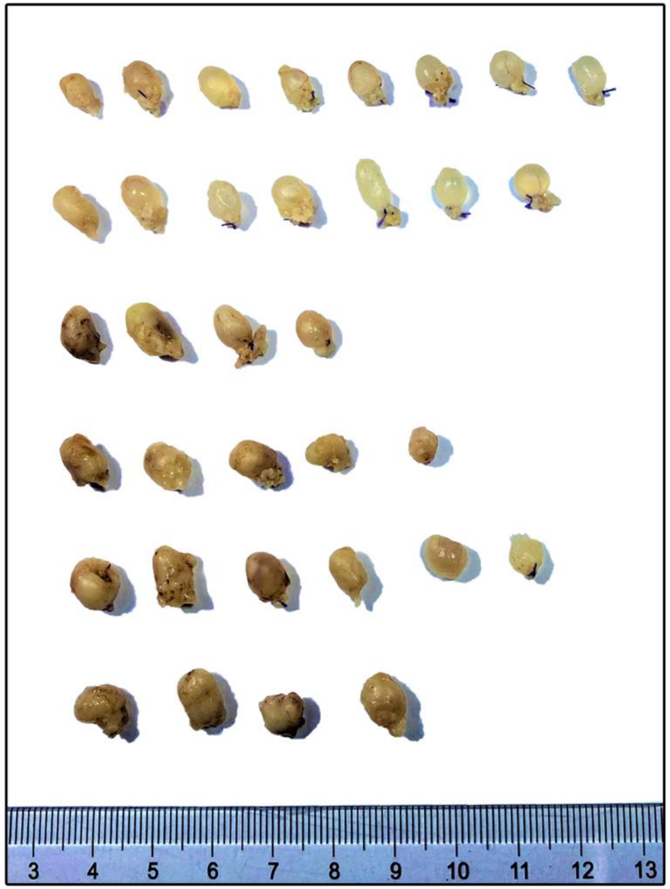

Figure S1. The bladders from the orthotopic bladder cancer models were dissected and fixed at the end of the study. Most of the bladders from the RC48-ADC-treated groups were free of tumours or had only small tumours when visualized with the naked eye. However, large tumours inside the bladder were observed in the disitamab, MMAE, and epirubicin treatment groups and the control group.

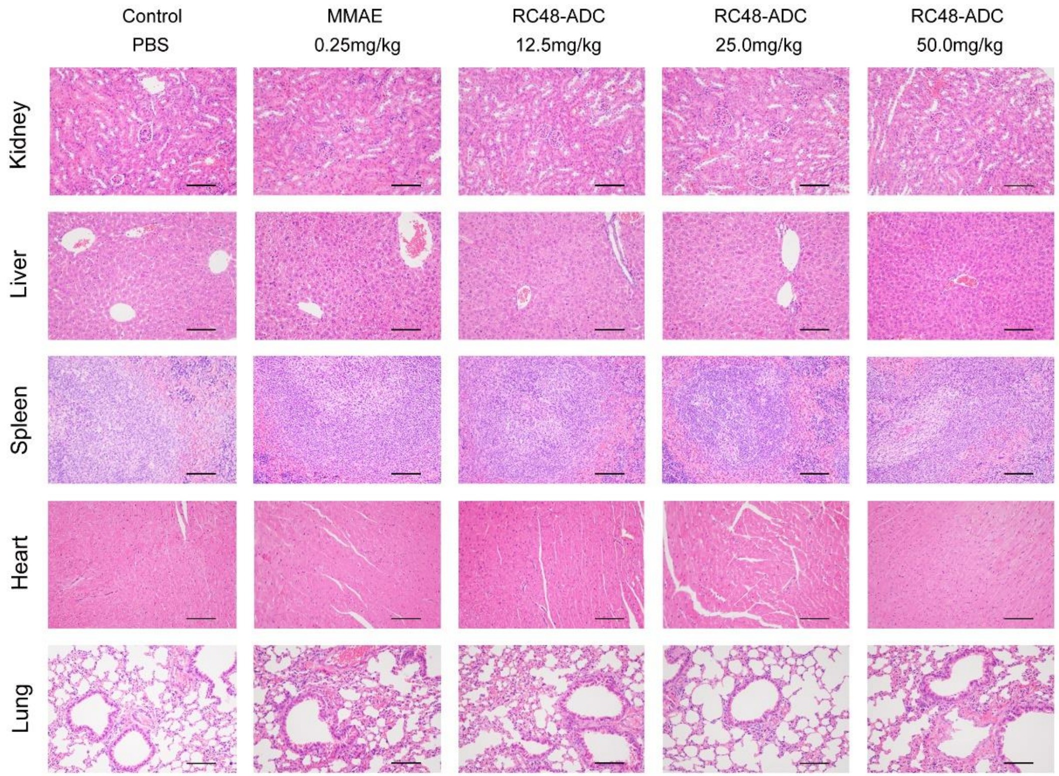

Figure S2. Representative images of H&E-stained slices of the major organs at the end of the repeated-dose toxicity study. Scale bars: 100 μm

(black).

HE staining of major organs showed that there were no significant histological changes in the kidney, liver, spleen, heart and lung of mice in each treatment group.

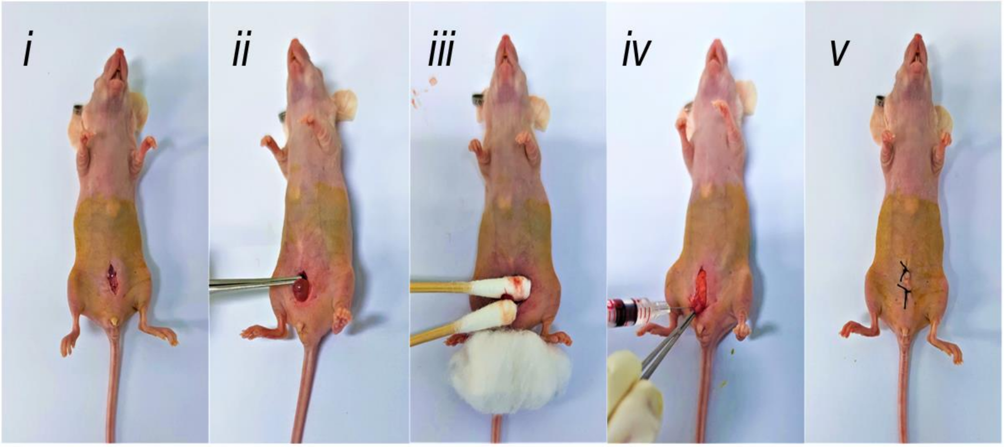

Figure S3. The surgical approach to constructing an orthotopic BCa model in mice.

2.0×10^6 luciferase-expressing T24 cells per mouse were prepared in 50 μl of RPMI 1640. After surgical site preparation, a 1 cm midline incision through the skin of the abdominopelvic region was made to expose and exteriorize the urinary bladder. Urine was squeezed slightly from the bladder using cotton swabs. A needle is gently inserted into the bladder base. After ensuring that the needle point was within the vesical lumen, the cancer cells were slowly injected. The needle point was gently clamped with forceps to prevent leakage of the cancer cell suspension. Then, the bladder is returned to the abdominopelvic cavity. Finally, the abdominal muscle and skin were sutured and closed.

Supplementary Tables

Table S1. In vitro cytotoxicity potency (IC_{50}) and maximum inhibition of RC48-ADC in comparison with other agents after 72 h exposure in various tumour cells

	RC48-ADC		Disitamab		MMAE		Epirubicin		Gemcitabine	
	IC_{50} (nM)	Inhibition (%)	IC_{50} (nM)	Inhibition (%)	IC_{50} (nM)	Inhibition (%)	IC_{50} (nM)	Inhibition (%)	IC_{50} (nM)	Inhibition (%)
5637	1.484 ± 0.435	83.49 ± 1.87	>1000	19.59 ± 1.55	0.160 ± 0.050	81.88 ± 1.04	171.1 ± 31.7	85.51 ± 0.98	130.9 ± 28.9	83.17 ± 1.20
T24	9.295 ± 2.524	85.94 ± 2.21	>1000	14.17 ± 1.75	1.515 ± 0.217	86.32 ± 0.78	153.8 ± 33.0	90.01 ± 0.72	647.6 ± 103.2	89.00 ± 1.97
UM-UC-3	221.9 ± 46.6	56.47 ± 1.62	>1000	8.22 ± 1.37	2.519 ± 0.661	71.55 ± 1.80	646.3 ± 58.5	70.74 ± 1.40	220.7 ± 60.4	71.57 ± 1.58
SK-BR-3	0.084 ± 0.030	87.00 ± 1.19	>1000	23.32 ± 0.61	0.847 ± 0.191	86.68 ± 1.03				
MDA-MB-231	459.7 ± 63.5	56.00 ± 3.56	>1000	4.10 ± 1.58	10.856 ± 1.291	83.17 ± 0.97				

Table S2. Effects of RC48-ADC on blood cells analysis and serum biochemical detection in the repeated-dose toxicity study.

	Control	MMAE 0.25 mg/kg		RC48-ADC 12.5 mg/kg		RC48-ADC 25.0 mg/kg		RC48-ADC 50.0 mg/kg	
	Mean±SD	Mean±SD	P value	Mean±SD	P value	Mean±SD	P value	Mean±SD	P value
WBC, 10^9/L	3.70±0.36	3.70±1.01	1.000	5.27±1.84	0.222	3.33±0.70	0.466	3.67±0.81	0.951
RBC, 10^{12}/L	9.27±1.28	8.96±0.67	0.729	9.92±0.26	0.433	9.97±1.01	0.495	9.71±1.06	0.671
PLT, 10^9/L	1167±89.31	1165.67±278.91	0.994	1111.67±210.18	0.696	934.67±244.24	0.197	959.40±208.14	0.188
CR, µmol/L	34.07±2.77	28.00±5.18	0.148	30.53±5.26	0.360	29.91±2.49	0.125	96.64±32.86	**0.030**
BUN, mg/dL	25.16±2.38	29.04±3.81	0.209	29.56±7.55	0.391	22.82±5.54	0.538	52.86±16.08	**0.042**
ALT, U/L	61.61±9.87	65.68±7.97	0.608	65.49±22.99	0.801	47.62±7.61	0.124	80.94±21.34	0.228
AST, U/L	147.17±39.67	180.02±67.75	0.509	168.66±45.26	0.570	138.69±48.39	0.826	194.74±71.89	0.372
ALB, g/L	33.75±1.25	35.08±1.15	0.246	35.57±0.68	0.091	35.03±3.21	0.555	27.86±4.16	0.078
LDH, mmol/L	674.19±313.79	523.99±233.06	0.542	637.23±341.60	0.897	453.87±146.41	0.332	538.96±83.62	0.511

Abbreviations: WBC=white blood cell count; RBC=red blood cell count; PLT=platelet; CR=creatinine; BUN=blood urea nitrogen; ALT= alanine aminotransferase; AST= aspartate aminotransferase; ALB=albumin; LDH= lactate dehydrogenase.

Table S3. Correlation between HER2 expression and clinicopathological characteristics of NMIBC patients from SYSMH Cohort.

Characteristic	Patient frequency	HER2				Spearman Correlation	p value
		0	1+	2+	3+		
Total	48 (100.0%)	6 (12.5%)	8 (16.7%)	27 (56.2%)	7 (14.6%)		
Age						0.178	0.220
≤65 years old	31 (64.6%)	5 (10.4%)	6 (12.5%)	16 (33.3%)	4 (8.3%)		
> 65 years old	17 (35.4%)	1 (2.1%)	2 (4.2%)	11 (22.9%)	3 (6.3%)		
Gender						0.077	0.610
Male	35 (72.9%)	5 (10.4%)	7 (14.6%)	17 (35.4%)	6 (12.5%)		
Female	13 (27.1%)	1 (2.1%)	1 (2.1%)	10 (20.8%)	1 (2.1%)		
T stage						0.377	**0.010**
Ta	21 (43.8%)	4 (8.3%)	5 (10.4%)	12 (25.0%)	0 (0%)		
T1	27 (56.2%)	2 (4.2%)	3 (6.3%)	15 (31.3%)	7 (14.6%)		
Tumor grade						0.404	**0.006**
Low	9 (18.8%)	3 (6.3%)	3 (6.3%)	3 (6.3%)	0 (0%)		
High	39 (81.2%)	3 (6.3%)	5 (10.4%)	24 (50.0%)	7 (14.6%)		
Disease stage						0.308	**0.036**
Initial diagnosis	33 (68.8%)	6 (12.5%)	6 (12.5%)	18 (37.5%)	3 (6.3%)		
Recurred disease	15 (31.2%)	0 (0%)	2 (4.2%)	9 (18.8%)	4 (8.3%)		

Histological classification						0.084	0.529
UC without divergent differentiation	44 (91.7%)	6 (12.5%)	8 (16.7%)	23 (47.9%)	7 (14.6%)		
UC with divergent differentiation	4 (8.3%)	0 (0%)	0 (0%)	4 (8.3%)	0 (0%)		

Abbreviations: UC=urothelium carcinoma

Table S4. Clinical information of human BCa organoids.

ID	Source	Age	Gender	Histological type	Histological grade	T stage	HER2 Score
PDO-1	TURBT	58	Male	UC	High grade	T1	2+
PDO-2	TURBT	62	Male	UC	High grade	T1	2+
PDO-3	TURBT	56	Male	UC	High grade	T1	2+
PDO-4	TURBT	63	Male	UC	High grade	T1	(-)
PDO-5	TURBT	38	Male	UC	Low grade	Ta	(-)
PDO-6	TURBT	57	Male	UC	High grade	Ta	(-)

Abbreviations: PDO=patient-derived organoid; TURBT=transurethral resection of bladder tumor; UC=urothelial carcinoma

Table S5. Contents of BCa organoids culture medium.

Reagent	Supplier	Cat. No.	Final concentration
Advanced D-MEM/F-12	ThermoFisher	12634010	1×

collagenase type II	Worthington	LS004176	5 mg/mL
DNAse I	Worthington	LS002138	10 µg/mL
ROCK inhibitor	Sigma	Y-27632	10 µM
Primocin	InvivoGen	ant-pm-05	100 µg/mL
GlutaMAX	GIBCO	35050061	1×
HEPES	GIBCO	15630080	1×
B-27TM Supplement	GIBCO	17504044	1×
N-Acetylcysteine	Sigma	A9165	1.25 mM
Nicotinamide	Sigma	N0636	10 mM
A83-01	Sigma	SML0788	500 nM
R-spondin1	NovoProtein	CX83	500 ng/mL
Noggin	NovoProtein	CB89	100 ng/mL
FGF-10	Peprotech	100-26	20 ng/mL
FGF-2	NovoProtein	C779	5 ng/mL
EGF	Peprotech	100-15	50 ng/mL

Table S6. Primers used in qPCR

Primer Name	Sequence 5'-3'
HER2 Forward	CCAGCTCTTTGAGGACAACTAT
HER2 Reverse	TTTCAAGATCTCTGTGAGGCTT
GAPDH Forward	CAATGACCCCTTCATTGACC
GAPDH Reverse	TTGATTTTGGAGGGATCTCG

Table S7. Primary antibodies used in this study

Antibody Name (application)	Cat No.	Brand
GAPDH (WB)	60004-1-Ig	Proteintech
HER2 (WB, IF)	18299-1-AP	Proteintech
CDK1 (WB)	10762-1-AP	Proteintech
Cyclin B1 (WB)	28603-1-AP	Proteintech
PARP (WB)	YT6210	Immunoway
Cleaved PARP (WB)	YC0101	Immunoway
Cleaved Caspase 3 (WB)	YC0006	Immunoway
Cleaved Caspase 7 (WB)	YC0010	Immunoway
Ki-67 (IHC)	YM6189	Immunoway
VENTANA HER2 (IHC)	4B5	Roche
